# c302: a multiscale framework for modelling the nervous system of *Caenorhabditis elegans*

**DOI:** 10.1098/rstb.2017.0379

**Published:** 2018-09-10

**Authors:** Padraig Gleeson, David Lung, Radu Grosu, Ramin Hasani, Stephen D. Larson

**Affiliations:** 1 Department of Neuroscience, Physiology and Pharmacology, University College London, London, UK; 2 Cyber-Physical Systems, Technische Universität Wien, Vienna, Austria; 3 OpenWorm Foundation, Boston, MA, USA

**Keywords:** simulation, *C. elegans*, computational neuroscience, standardization, open source

## Abstract

The OpenWorm project has the ambitious goal of producing a highly detailed *in silico* model of the nematode *Caenorhabditis elegans*. A crucial part of this work will be a model of the nervous system encompassing all known cell types and connections. The appropriate level of biophysical detail required in the neuronal model to reproduce observed high-level behaviours in the worm has yet to be determined. For this reason, we have developed a framework, c302, that allows different instances of neuronal networks to be generated incorporating varying levels of anatomical and physiological detail, which can be investigated and refined independently or linked to other tools developed in the OpenWorm modelling toolchain.

This article is part of a discussion meeting issue ‘Connectome to behaviour: modelling *C. elegans* at cellular resolution’.

## 1. Introduction

Computational models of the nervous system are developed at multiple scales to answer questions about how low level interactions between biological entities lead to higher level functions [1–3]. Models based on the nematode *Caenorhabditis elegans* have been created at many levels including individual neurons [4] and muscles [5], subcircuits responsible for generating specific behaviours [6–9], body-wide processes including locomotion [10–12], and detailed nervous system/musculature models [13]. Each of these models selects a subset of anatomical and physiological properties known to exist in the worm and can address a specific set of questions relevant to that level of detail. However, simplifying assumptions are often made to facilitate theoretical analysis of the model or for computational efficiency, and this can often lead to difficulty using the developed model for addressing other questions outside the scope of the original study [14].

The OpenWorm project [15] aims to create an open source computational model of *C. elegans* which will be constrained by biological data down to the individual cell level, and can be used to simulate the behaviour of the worm as it interacts in a 3D environment. While a fully constrained, highly detailed model of the worm reproducing a range of known behaviours requires much further work, a number of subprojects have been developing the computational infrastructure required for such detailed simulations (see also [16,17] in this issue). An important driving factor in these subprojects has been ensuring the individual applications are useful as research tools in their own right, independent of other elements of the OpenWorm platform and in scenarios where the full-scale worm model would not be appropriate for the scientific question being addressed.

An essential part of the detailed worm model developed by the OpenWorm project will be a simulation of the nervous system of *C. elegans*, which will have to interact with all other elements of the platform. To this end, a computational framework in the Python scripting language, c302, has been developed which aims to facilitate the creation of models of the nervous system and musculature of *C. elegans*. Models generated by the framework can contain all known cells or subsets thereof, and can have varying levels of biophysical detail for the individual neurons, muscles and synapses. Information on the numbers, types and polarity of synaptic connections are incorporated into the models from the structured information on these gathered by the OpenWorm project.

The c302 models are generated in standardized format (NeuroML [18,19]) ensuring they can be used with a variety of preexisting tools and libraries for model visualization, simulation and analysis. This will facilitate models generated by c302 being incorporated into the detailed 3D worm body models in OpenWorm. It will allow simulation of network activity during behaviour down to the level of the membrane potential and internal calcium dynamics of individual neurons, while also allowing more abstract neuronal models to be used where appropriate. This open source framework can be used for multiple types of investigation into the dynamical underpinnings of the *C. elegans* nervous system.

## 2. Methods

### (a) NeuroML

The elements of the neuronal networks that are generated by this framework are expressed using the NeuroML model description language [18,19]. NeuroML is an XML-based format which specifies the hierarchical structure and parameters required to create models of cells, ion channels, synapses, input stimuli and 3D populations of synaptically connected neurons. Models described in this format can be parsed by or mapped to the native formats of neuronal simulation platforms to simulate the dynamical behaviour of the systems described. There are a growing number of applications, libraries and databases which support NeuroML, allowing *C. elegans* models in this format to be more easily visualized, analysed and compared with other models, using a number of pre-existing tools.^1^ The options for specifying cell models in NeuroML format range from abstract point-neuron models with one (e.g. leaky integrate and fire, LIF) or two state variables [20,21], to conductance-based models with active membrane currents, which can have a single compartment, a stylized representation for dendritic and axonal trees or be based on detailed reconstructions of neuronal morphologies. Ion channel models can be based on the Hodgkin–Huxley formalism [22] or kinetic scheme/Markov models [23]. In addition to the electrical behaviour of the modelled cells, an internal pool of calcium can be represented, as well as its influence on the conductance of calcium gated ion channels. Synapse models include spike triggered chemical synapses (either fixed or plastic based on activation history), continuously transmitting analogue synapses, or electrical transmission through gap junctions.

An important feature of the NeuroML language introduced in version 2 [19] is that model structure and dynamics are specified in a machine readable format, LEMS (Low Entropy Model Specification language). This describes the hierarchical structure of the model elements, but also the dimensional parameters for each element, and how the state variables change, both continuously and in response to events in the network (e.g. a NeuroML file for a LIF cell will only state the value of the threshold and reset voltages, capacitance, etc. but the LEMS definition will state how to calculate the membrane potential from these parameters at each time step). This unambiguous format for the dynamical behaviour of the model allows simulators to be developed which natively understand the LEMS format and can simulate the models, as well as facilitates the mapping to other simulation platforms. While a number of widely used model types are present in the core NeuroML elements, these can easily be extended with new cell, channel or synapse models by creating definitions in LEMS, which are then compatible with the rest of the model generation and simulation toolchain.

### (b) Python

The programming language in which the c302 framework is built is Python, and it is through scripts in this language that users can interact with it. Many software applications in computational neuroscience have added scripting interfaces for Python in recent years [24,25], and the open source nature of the language and associated modules has made it the chosen language for a number of subprojects in the OpenWorm initiative. For example, the package PyOpenWorm^2^ gives programmatic access via Python scripts to numerous types of data on *C. elegans*, as can be found in resources such WormBase [26] and WormAtlas.org.^3^

A number of Python modules have been developed to support models in NeuroML and LEMS. libNeuroML^4^ [27] can be used to parse, edit, save and validate NeuroML documents, while PyLEMS^5^ is a Python module for reading/writing LEMS files, while natively allowing simulation of the majority of LEMS models. pyNeuroML^6^ builds on libNeuroML and gives access to a greater range of functionality for handling NeuroML inside Python scripts, and crucially allows access to all of the functionality originally developed in Java [19] for converting NeuroML into code for dedicated high-performance neuronal simulators (e.g. NEURON [28]).

### (c) Neuronal simulations

Simulation of the electrical activity is a crucial use case for the generated network models. jNeuroML is a Java implementation of LEMS [19] and can run simulations of any of the described networks where the neuron models are represented by a single compartment. NEURON [28] is a neuronal simulator which is widely used in the computational neuroscience community, including for the Blue Brain Project neocortical microcircuit model [29] and Allen Institute MindScope project [30]. All networks generated in c302 can run in NEURON, and simulations are generally faster than in jNeuroML.

Simulation of the internal calcium concentration dynamics is also important for modelling the nervous system of *C. elegans*, as it allows comparison against the growing body of calcium imaging results [4,31–33]. This type of modelling is well supported in both jNeuroML and NEURON.

### (d) Open Source Brain

Open Source Brain (OSB, [34]) is an online resource promoting the collaborative development and sharing of standardized models in computational neuroscience.^7^ Users of OSB can create projects linked to open source code sharing repositories (e.g. on GitHub^8^) containing NeuroML models, which can then be accessed and visualized in 3D on OSB. Model cell and network properties can be analysed, and simulations generated (by converting the NeuroML files into simulator specific code), executed on the OSB servers and replayed inside the browser. A core component of OSB for handling the NeuroML models is Geppetto^9^ [16], a Web-based platform for model visualization and simulation which was originally developed as part of the OpenWorm project.

### (e) Cell models

Incorporating previously published model elements into the c302 framework will be an important part of ensuring the resultant network is constrained at many levels. Two published cell models from *C. elegans* have been converted to NeuroML and can be included in c302 networks. The muscle cell model of Boyle & Cohen [5] includes fast and slow K^+^ currents and an inactivating Ca^2+^ current.^10^ Another muscle model [35] with one K^+^ and one Ca^2+^ channel has recently been incorporated into the framework.^11^ Both of these models are based on experimental work of Jospin and colleagues characterizing the K^+^ and Ca^2+^ currents in the body wall muscle of *C. elegans* [36,37].

### (f) Synapse models

While the majority of chemical synapse models used in network models of vertebrate systems are spike triggered (using the crossing of a specific threshold in the presynaptic cell as a signal for a transient change in postsynaptic conductance), a continuously transmitting, analogue synapse is a better approximation of synaptic transmission for *C. elegans* [38]. To this end, two options for analogue synapses have been implemented for use in c302, one based on a model developed for the pyloric network of the crustacean stomatogastric ganglion [39] and another from a network model of *C. elegans* locomotion with simple passive neurons but continuously transmitting synapses [11].

### (g) Availability of software

All of the software packages described here are open source and available for download. The main framework for c302 can be found at https://github.com/openworm/c302. The README file for the package gives installation instructions as well as examples of how to run the standard examples, generate new networks, change parameters in the configurations, add new stimulations, and visualize and run the networks on OSB. The Python package can also be installed using *pip install c302*. A Docker^12^ image containing c302 and a number of other OpenWorm packages configured to work together is available at https://github.com/openworm/OpenWorm.

## 3. Results

### (a) c302

**Figure 1.**
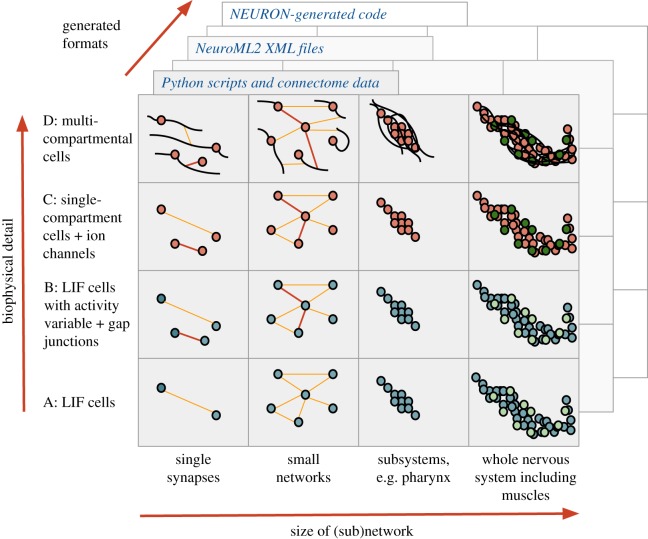
c302 overview. The increasing levels of biophysical detail that can be used for models generated by the framework are shown (A*–*D on *y*-axis), along with examples of subnetworks of the worm's neuromuscular system which are to be created (*x*-axis). Each of the 16 boxes represents a generated instance of cells (small circles for somas with black dendrites/axons where present) connected by chemical (orange) or electrical (red) synapses. The neurons can be represented by LIF- (blue) or conductance-based (dark orange) models, as can the muscle cells (light/dark green). Specific instances of the network can be generated by Python scripts, which save the model structure as NeuroML. This can in turn be automatically converted to supported formats, including NEURON to simulate the electrical activity of the model.

The framework we developed, c302, allows the generation of a wide range of network models, which can be used to investigate different aspects of the *C. elegans* nervous system. c302 makes the processes of code generation, network simulation and analysis as transparent as possible for the user. It breaks down the space of all possible network configurations along two broad axes (figure 1): the subset of the neuronal network to incorporate; and the level of detail to use in the individual neurons and synapses. Use of the framework involves specifying a configuration along each of these axes, and the relevant files for simulating the corresponding network can be created.

**Figure 2.**
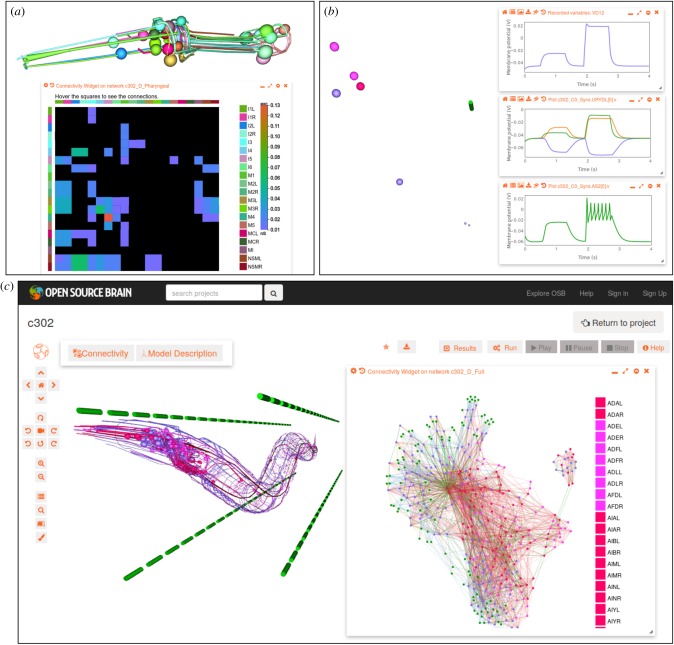
c302–generated models visualized on Open Source Brain (OSB) website. (*a*) Screenshot of network of 20 neurons present in the pharynx of *C. elegans*. Spherical somas and dendrites/axons of the cells can be seen on top (each cell has different colour). Window on bottom shows interactive connectivity matrix, with bars on left/top of main matrix showing colour corresponding to pre/postsynaptic cells respectively (cells are defined on right of matrix). Each block in the matrix is coloured by the weight of the chemical connections based on number of known connections from the connectome (black: no connection; purple: one connection/0.01 nS; red: 13 connections, 0.13 nS). (*b*) Network with four pairs of synaptically connected cells (left; neurons in purple, pink and red, muscle in green; cells have same 3D locations as used for corresponding somas in *c*). Membrane potential plots from simulation executed via OSB Web interface are shown on right. In all cases the presynaptic neuron receives two pulses of input (top plot) and the response of neurons connected via excitatory chemical synapse (orange), inhibitory chemical synapse (purple) and gap junction (green) are shown in middle plot. Bottom plot shows response of muscle cell connected via excitatory synapse. (*c*) Screenshot of OSB project page for c302 showing network containing all neurons and muscles. Neurons are coloured according to type (red: interneurons; pink: sensory; purple: motor neurons) and the four quadrants of muscles (green) are located away from the body for clarity. Window on right shows connections in network in a 2D force-directed graph (colours of circles for cells correspond to those in 3D view). Cells with stronger connections are located closer together. The 20 cells of the pharynx are clearly separated on the right owing to lower numbers of connections from these to other cells in the rest of the network. Green muscle cells are also clearly visible on the periphery. Not all cells are listed on right, but hovering over individual circles in the Web browser will show the name of the cell.

The framework allows all or subsets of the 302 neurons of the adult hermaphrodite to be included in the model (figure 1, *x*-axis). While the level of detail of the model of the neuron (single or multicompartmental, see below) determines whether the full 3D structure of the axons and dendrites are included, in all cases the somas of the relevant neurons are positioned at the known location for that cell. The cellular reconstructions of Christian Grove as part of the Virtual Worm project [40] were used as the basis of the multicompartmental NeuroML models. Additional data from PyOpenWorm on cell classes (sensory, motor neuron, interneuron, etc.), receptors and neurotransmitters are added to the cell models on generation.^13^ Muscle cells can also be generated and these are organized into four quadrants, each containing 24 muscles for convenience.^14^ These muscles are positioned in four rows separated from the worm body to facilitate visualization of connections/activity (as shown in figure 2*c*).

Connectivity parameters between neurons (and between neurons and muscles) can be taken from two alternate sources: (i) spreadsheets generated by Varshney and colleagues [41] and obtained from WormAtlas^15^; (ii) data generated by the WormWiring project^16^ of the Emmons laboratory at Albert Einstein College of Medicine. The latter source, while more complete, is not yet published, and the former source may be more appropriate for many studies. In any case, the network can be regenerated with either connectome by changing a single parameter in the configuration script.

Regarding the level of biophysical detail that can be selected for the neurons and synapses, a number of sample configurations have been created (figure 1, *y*-axis). Each configuration describes the cell model to use (based on NeuroML prototypes; currently all neurons are based on one set of cell parameters, and all muscles are based on another set), and the parameters for the excitatory chemical synapses (defined in this first approximation as those where the neurotransmitter of the presynaptic cell is not GABA), inhibitory chemical synapses (GABA-transmitting cells), and gap junctions. By default, individual connections between pairs of cells within each of these three classes of connections differ only in their weights (the product of a baseline conductance and the known number of connections between the pair taken from the selected connectome). However, the weights of individual connections can be modified to investigate how such changes impact network behaviour.

A number of parameter sets (fully specifying the NeuroML models at that level of detail) have been created which vary from simplistic representations of the network to biophysically detailed representations (figure 1, *y*-axis). These are parameters A (comprising LIF cells; spike triggered synaptic transmission for all connections); parameters B (LIF cells with an extra parameter based on cell firing rate, as a proxy of cell activity; electrical connections for gap junctions); parameters C (single-compartment, conductance-based cells with ion channels; spike triggered synapses and gap junctions); parameters D (similar to C with multicompartmental cells). Some variations of these parameter sets are included with the c302 framework; for example C1 and D1 (based on parameter sets C and D respectively) use analogue synaptic connections for chemical synapses.

Python scripts describe each of the network subsets (e.g. *c302_Pharyngeal.py, c302_Full.py*) and each parameter set (*parameters_A.py*, etc.). Generation of a NeuroML description of one of the 16 configurations shown in figure 1 only requires specifying the subset of the network and a biophysical detail parameter set; for example *python c302_Pharyngeal.py D* generates the network illustrated in figure 2*a*.

The code repository for c302 has been linked to an OSB project.^17^ Figure 2 shows a number of generated models displayed on OSB. Figure 2*a* illustrates 20 cells from the pharynx generated with full morphologies, along with a connectivity matrix for the network. Figure 2*b* shows the result of running a simulation via OSB containing eight single-compartment cells. Plots are shown of the membrane potentials recorded from the cells. A network with the full complement of neurons and muscles is shown in figure 2*c*. The inset shows a visualization generated from the network connections which have been extracted from the NeuroML description.

### (b) Case study of network model in c302: forward locomotion

To illustrate the ability of the c302 framework to generate network models exhibiting realistic behaviours, we developed an example network incorporating single-compartment, conductance-based neurons and muscles (based on parameters C1), and a subset of neurons known to be involved in forward locomotion, to investigate whether the network produces muscle activity that can be used to drive the worm forwards.

**Figure 3.**
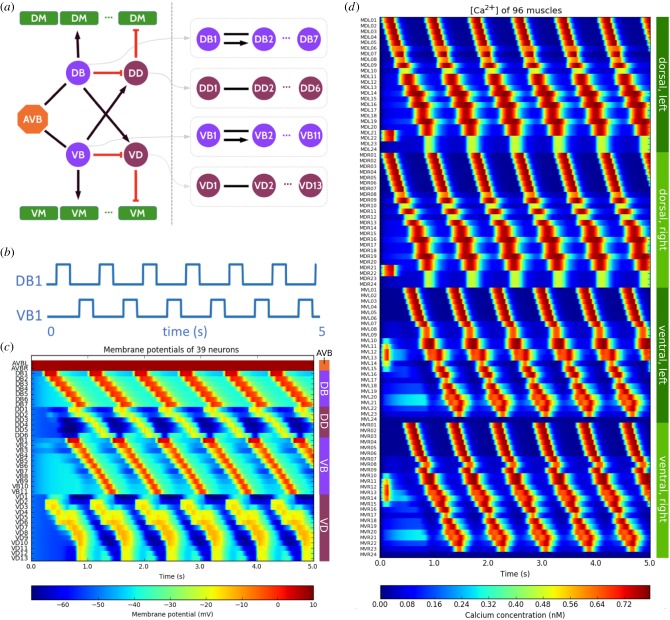
Simulation of neuronal and muscle activity of *C. elegans* during forward crawling. (*a*) Symbolic representation of the neural circuit composed of AVB interneurons, B-type and D-type motor neurons for generation of the forward crawling activity. Gap junction connections represented by black lines, excitatory chemical synapses by black arrows, inhibitory synapses by red connections. On the left, a high-level view of connectivity between classes of neurons and the dorsal (DM) and ventral (VM) muscle groups is shown. On the right, connections between the individual neurons within each of the DB, DD, VB and VD classes are illustrated (the dots indicate the same connections as between cells 1 and 2 are present between 2 and 3, and so on). (*b*) Hypothetical central pattern generator modulatory inputs to DB1 and VB1 motor neurons. (*c*) Motor neuron activity during 5 s of real-time simulation of the forward locomotion neural circuit. (*d*) Activity of the body-wall muscles (variations in [Ca^2+^]) during the forward locomotion simulation.

The circuit (figure 3*a*) comprises the following neurons: left and right pair command neurons AVB (AVBL, AVBR), 18 B-type motor neurons including 7 dorsal (DB1–DB7) and 11 ventral (VB1–VB11), 19 inhibitory D-type motor neurons consisting of 6 dorsal (DD1–DD6) and 13 ventral (VD1–VD13), and 96 body-wall muscle cells (with only 95 receiving input). We used the wiring data generated for the hermaphrodite *C. elegans* by the WormWiring project, apart from the connections that have been overwritten below. As the weights of the synaptic connections in the *C. elegans* connectome have not yet been defined [42], we simplified the network by assuming all synaptic connections share the same weight in the network, even if multiple individual connections are present in the connectome.

To generate the activity required for forward locomotion, we used the following simplifying assumptions when constructing the network:

— *Assumptions on the network structure*. A symbolic representation of the hypothesized circuit for simulating forward crawling is shown in figure 3*a*. AVB makes gap junctions with B-type motor neurons. We assume that DB and VB, cholinergic motor neurons, excite their downstream muscle cells with excitatory synapses while GABAergic neurons, DD and VD groups, inhibit the muscle cells. DB group excites VD neurons and inhibits the DD motor neurons. Similarly, VB motor neurons activate the DD inhibitory motor neurons and inhibit the VD motor neurons. These hypotheses for the polarity of synapses in the network are preliminary assumptions to create the forward locomotion activity, which obviously require further experimental validation.
— *Head muscle cells are directly stimulated by synchronized periodic current pulses*. Neurons that are presynaptic to the head muscle cells are not included in the model. We therefore directly injected synchronized oscillatory current pulses to generate alternating bends of the dorsal and ventral muscles in the head (the first seven muscle cells in each group: left/right dorsal muscles, left/right ventral muscles). We adjusted the delays between dorsal and ventral muscles and between two dorsal/ventral pulses so that the muscles of the head contract with the same frequency as the rest of the body.
— *AVB neurons are active during the forward crawling period*. In a forward movement state, AVB neurons are active [32]. They modulate locomotion of the worm by inducing or accelerating forward movement. Synaptic inputs to the AVB neuron pairs from their upstream neurons were approximated by an input current pulse into the cell for the duration of the simulation, in order to keep the neuron active during the forward-movement period.
— *An external current pulse, hypothesized as a central pattern generator system, periodically stimulates the first dorsal and ventral B-type motor-neurons*. We assumed a central pattern generator (CPG) mechanism that induces phase-shifted dorsoventral body bends in the B-type motor neuron networks, from the neck posteriorly to the tail. We approximated the input from this hypothetical CPG by injecting periodic current pulses directly into the first B motor neurons, DB1 and VB1 (figure 3*b*). These currents then flow through the chain of B-type motor neurons which are linked to each other with gap junctions.
— *A proprioceptive mechanism in B-type motor neurons*. When a body segment bends, a posteriorly located motor neuron receives additional excitatory current due to stretch receptors signalling the bending of a more anterior body segment [43]. As a result of such a proprioceptive mechanism, the bends get propagated along the body. We added a functional excitatory mechanism between neighbouring DB/VB neurons to approximate the propagation of this proprioceptive feedback. This is symbolically shown in (figure 3*a*, right) by an arrow between the B-type motor neuron groups.

The forward locomotion cell network was simulated within the c302 framework for 5 s. Figure 3*c*,*d* respectively represents the membrane potential dynamics of the individual neurons, and the intracellular calcium kinetics of all 96 body muscles (note that MVL24 is silent). The simplified circuit successfully generated travelling waves in the muscle cells, from head to tail, which are observed experimentally during forward locomotion [44].

**Figure 4.**
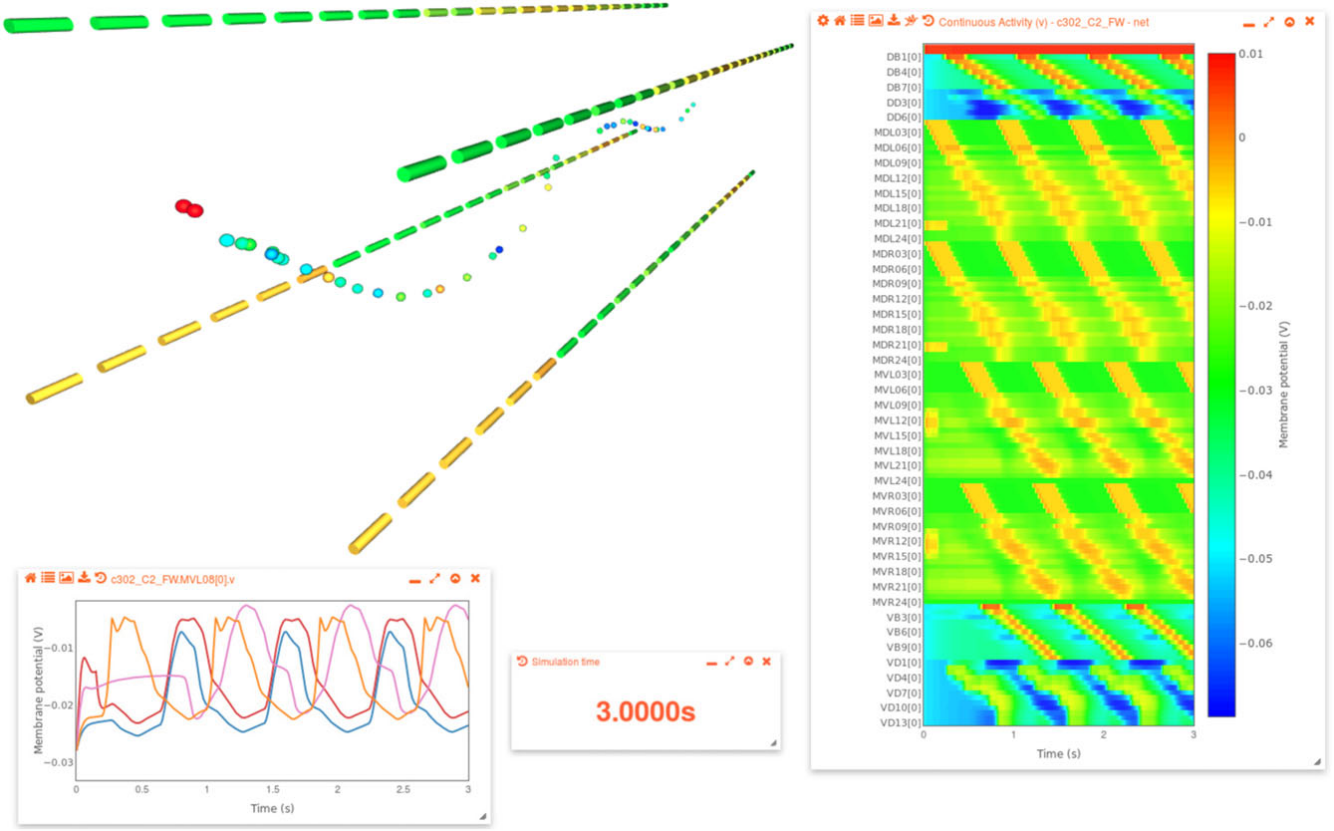
Activity of forward locomotion network in c302 simulated and visualized on OSB. 3D image on top left shows the 39 neurons that are modelled as single compartments following the line of the worm body, along with the four muscle quadrants (as shown in figure 3*c*). Window on right shows the membrane potential of all cells as a heatmap. Cells are arranged in alphabetical order from top to bottom and a subset of the cell names are shown on the left of heatmap, with a scale on the right. Window on the bottom left shows a selection of membrane potential traces of muscles, and the current time of the simulation replay is also displayed. The scale on the heatmap is also used for the colouring for the 3D cells, which changes with time as the saved simulation results are replayed. The approximately synchronous activation of the two dorsal muscle quadrants, and out-of-phase activity of the ventral quadrants, can be seen in this 3D view.

The generated network can be visualized on OSB along with the activity of the cells (figure 4). In this way, simulations of the network can be set running, parameters edited and changes in activity visualized, all from within the browser.

## 4. Discussion

We developed a framework in Python that can be used to generate a number of configurations of network models for investigating the activity of the nervous system of *C. elegans*. Users select the level of biophysical detail to use for the cells and synapses, together with the subset of cells to use in the network, and the framework will generate the network expressed in standardized NeuroML format, which can be used for visualizing, analysing and simulating the network. To illustrate the functionality of the framework, we developed a network model comprising a subset of neurons involved in forward locomotion together with the four muscle quadrants, and showed how this network of conductance-based, single-compartment cell models can give rise to travelling waves of activation in the muscles, which would be required for moving the worm forwards.

There has been much previous work in simulating individual elements [4,5,35] or network models of *C. elegans* [6,9–12]. While many useful insights have been gained by these individual studies, the computational models themselves are usually difficult to reuse for other investigations, and may be quite difficult to reproduce if the original source code has not been made available [45]. The c302 framework has been developed to create a generic platform on which multiple studies can be carried out, and with an ecosystem of tools to greatly facilitate disseminating, comparing and reusing models. The special advantage of doing this for *C. elegans* is that its individual neurons have been named and many connections and relationships well studied, and models in this space already have a high degree of conceptual overlap, compared with models in other neuronal systems where there is far less consensus about the important level of modelling abstraction among experimentalists*.* Making the source code for c302 available from the start has been a key aspect of ensuring maximal usability of the framework by others.

Use of a standard format, NeuroML, for generating networks comes with a number of advantages. Tools and libraries already exist that support NeuroML, and a platform like OSB opens up a range of options for visualizing, analysing and simulating the networks, and making them more accessible to non-computational neuroscientists. There are benefits in the other direction as well; the requirement for analogue synapses in c302 was the motivation for introducing this element into NeuroML, meaning other computational models using this synapse type could be converted to the format, e.g. the pyloric pacemaker network [39].

The c302 framework will be used to develop a highly detailed, well-constrained model of the nervous system to drive the 3D worm body simulation created using the Sibernetic fluid mechanics simulator [17,46], and to examine the parameter space under which the model exhibits realistic behaviours. However, it is not yet clear what level of detail in the nervous system will be required to achieve such a correspondence, so flexibly enabling more or less complex elements to be substituted into the network at each level will be important for this development process. It is also clear that many questions about nervous system function in *C. elegans* can be addressed along the way with simplified networks (as shown by the network presented here exhibiting basic properties required for forward locomotion), which can be gradually refined and assumptions/simplifications removed.

Initial work has already taken place to use the simulated [Ca^2+^] levels in the muscle cells of c302 (figure 3*d*) to drive the contraction of the corresponding muscles in the Sibernetic model. The resultant behaviour of the worm as it interacts with the surrounding fluid environment can then be investigated in terms of the neuronal properties. Since the actual contraction of the worm is calculated (as opposed to the expected bending based on the propagating oscillatory activity along the body) the system can be used for more accurate models of proprioceptive feedback. This will be important in investigating the various theories of how activity is generated and propagated along the body of the worm during locomotion [47]. Having full access to the 3D environment will also facilitate simulations of response to external stimuli such as touch and chemical or temperature gradients.

The c302 framework will also be extended to incorporate other known features of the *C. elegans* nervous system not currently supported. It is known that neurons can communicate through volume transmission of monoamines and neuropeptides [48]. These data have already been incorporated into PyOpenWorm, and therefore c302 is in a good position to incorporate the interaction networks implied by the data for a more complete description of neuronal information processing. The existing framework for modelling synaptic transmission through analogue synapses can readily be extended to handle such interactions. Individual biochemical signalling pathways and even gene expression networks could also be incorporated at the subcellular level via the existing mapping between NeuroML/LEMS and the Systems Biology Markup Language (SBML, [49]).

Other OpenWorm tools currently under development that will play an important part in improving and constraining c302 models include ChannelWorm,^18^ which aims to create a database of information on, and models of, ion channels known to be expressed in *C. elegans*; a SciUnit-based testing framework,^19^ to ensure model elements at different levels in the generated models conform to expected behaviour; and the Movement Validation subproject, which is producing specifications for, and an implementation of, the WCON (Worm tracker Commons Object Notation) format,^20^ which can be used to compare tracked worm movement as extracted from videos of behavioural experiments to simulated activity.

We hope that the c302 framework, together with the other tools in the OpenWorm toolchain, will form an accessible, open source platform on which to build and share detailed investigations into the neuronal underpinnings of *C. elegans* behaviour.

## Endnotes

1 See https://neuroml.org/tool_support.

2 See https://github.com/openworm/PyOpenWorm.

3 See https://pyopenworm.readthedocs.io/en/latest/data_sources.html.

4 See https://github.com/NeuralEnsemble/libNeuroML.

5 See https://github.com/LEMS/pylems.

6 See https://github.com/NeuroML/pyNeuroML.

7 See http://www.opensourcebrain.org.

8 See http://www.github.com.

9 See http://www.geppetto.org.

10 See https://github.com/openworm/muscle_model.

11 See https://github.com/openworm/JohnsonMailler_MuscleModel.

12 See https://www.docker.com.

13 See https://pyopenworm.readthedocs.io/en/latest/data_sources.html.

14 No connections are made to muscle ‘MVL24’ (the ventral left hand quadrant in the real worm has only 23 muscles) and so it never gets activated in the generated models.

15 See http://www.wormatlas.org/neuronalwiring.html.

16 See http://wormwiring.org/sex/hermaphrodite.php.

17 See http://www.opensourcebrain.org/projects/c302.

18 See https://github.com/openworm/ChannelWorm.

19 See https://github.com/openworm/tests.

20 See https://github.com/openworm/tracker-commons.

## Data accessibility

The software described in this article is open source and section 2g outlines how to access it.
